# Mechanisms by which in vitro meiotic arrest and sexual maturity improve developmental potential of mouse oocytes

**DOI:** 10.1038/s41598-017-16119-5

**Published:** 2017-11-17

**Authors:** Fei Chen, Juan Lin, Xue Sun, Bin Xiao, Shu-Fen Ning, Shuai Zhu, Hui-Li Wang, Jing-He Tan

**Affiliations:** 1 College of Life Science, Northeast Agricultural University, Harbin, 150030 P. R. China; 2 College of Animal Science and Veterinary Medicine, Shandong Agricultural University, Tai-an City, 271018 P. R. China

## Abstract

To study the relationship between chromatin condensation, gene transcription and developmental competence during oocyte maturation and to explore the mechanisms by which meiotic arrest maintenance (MAM) and sexual maturity improve oocyte competence, we examined effects of MAM with roscovitine or db-cAMP on chromatin condensation, gene transcription and developmental potential of NSN or SN oocytes from prepubertal or adult mice. MAM with roscovitine improved the developmental competence and global gene transcription of prepubertal NSN (prep-NSN) and adult-SN oocytes while having no effect on those of prep-SN oocytes. MAM with db-cAMP facilitated neither development nor transcription in any type of oocytes. MAM with either roscovitine or db-cAMP promoted chromatin condensation of prep-NSN oocytes. MAM with roscovitine promoted gene transcription and chromatin condensation simultaneously through inhibiting cyclin-dependent kinase (CDK) 5 and 2, respectively. The results suggested that MAM with roscovitine improved oocyte competence by promoting gene transcription via inhibiting CDK5. Oocyte cytoplasmic maturation is correlated with gene transcription but not with chromatin condensation. The difference in developmental competence between prepubertal NSN and SN oocytes and between prepubertal and adult SN oocytes was because while the former had not, the latter had completed or acquired the ability for transcription of important genes.

## Introduction

It is known that the developmental potential of the *in vitro* matured (IVM) oocytes is inferior to that of the *in vivo* matured ones. To gain the capacity to maintain successful embryo development, oocytes must undergo both nuclear and cytoplasmic maturation^1^. While oocytes acquire full cytoplasmic maturity *in vivo* after a long preparatory process involving transcription and translation of transcripts during the meiotic prophase^1,2^, a sudden premature meiotic resumption occurs *in vitro* without adequate cytoplasmic maturation following transfer of oocytes from follicles into the culture medium. Many studies have been conducted to improve oocyte cytoplasmic maturation through meiotic arrest maintenance (MAM) *in vitro*, but limited progress was achieved due to our limited understanding of the ongoing cellular activities during MAM. Although a recent study reported that oocyte maturation was significantly improved after MAM of oocytes from juvenile mice^3^, the underlying mechanisms are largely unknown.

The fully-grown germinal vesicle (GV) stage mouse oocytes are grouped into NSN (non-surrounded nucleolus) and SN (surrounded nucleolus) classes based on their chromatin configuration^4,5^. Oocyte competence appears to be influenced mainly by epigenetic factors that control gene expression and modify the GV chromatin configuration^6,7^. Thus, fully-grown GV oocytes must end a NSN configuration before gaining full meiotic competence, and they must take on a SN configuration and stop gene transcription before having the capacity to sustain blastocyst formation^7^. However, the factors underlying the differences in developmental competence between NSN and SN oocytes are not fully characterized. Furthermore, reports indicate that upon liberation from follicles, the NSN oocytes have a limited capacity to undergo spontaneous maturation to the metaphase II (MII) stage^5,8,9^, and that the NSN oocytes must metamorphose into SN ones during follicullogenesis to guarantee full-term embryonic development^4,6,10^. Thus, whether the NSN oocytes have the full capacity to accomplish nuclear maturation and whether the SN-stage is a prerequisite for cytoplasmic maturation remain to be determined.

Although studies have suggested that oocytes must take on a SN chromatin configuration and cease gene transcription before becoming capable of blastocyst formation^7,11,12^, the GV chromatin of maturing oocytes is synchronized in a less condensed state prior to GVBD in some species^7^. In bovine oocytes, for example, the GV chromatin was synchronized in a less condensed F pattern with floccular chromatin in the vicinity of the nucleoli and the nuclear envelope^13^, and mRNA synthesis was observed just before GVBD^14,15^. The preovulatory GV oocytes in mice^16–19^ and humans^20,21^ have also been reported to incorporate [^3^H] uridine. We recently observed that during maturation culture of pig oocytes, condensed chromatin was re-decondensed and gene transcription was evident prior to GVBD in some of the SN oocytes^22^. However, the prevalence of the pre-GVBD chromatin re-decondensation (RDC) and gene transcription has yet to be verified in other species and its significance and mechanisms are largely unexplored. Furthermore, although it is well known that oocyte developmental competence is lower in prepubertal animals than in adult animals^23–25^, the underlying mechanisms are largely unclear.

Roscovitine has been widely used for oocyte *in vitro* MAM in different species but different results were reported by different authors. For example, although all found the treatment of oocytes with roscovitine reversible, while some reported that treatment with roscovitine at optimal concentrations for optimal durations did not affect the developmental competence of treated oocytes in cattle^26^, goats^27^ and sheep^28^, others observed that the treatment had significantly detrimental effects in pigs^29^ and cats^30^ or markedly beneficial effects in goat oocytes from small follicles^27^ and in mouse oocytes^31^ on subsequent embryo development. Furthermore, although chromatin condensation was observed during MAM with butyrolactone I or roscovitine in bovine^32^, porcine^33^ and goat oocytes^27^, an increased gene transcription was evident during MAM of pig oocytes with roscovitine^22^. Thus, mechanistic studies are urgently needed for the differential effects of MAM with roscovitine on oocyte competence and for the contradiction between chromatin condensation and gene transcription during MAM with roscovitine or butyrolactone I.

In summary, the above literature review has revealed the following issues that remain to be addressed: (a) the limited knowledge on the ongoing cellular activities during MAM, such as gene transcription; (b) the mechanisms for the differential developmental competence between SN and NSN oocytes and between prepubertal and adult oocytes; (c) whether the NSN oocytes can complete nuclear maturation *in vitro* and if oocytes have to go through the SN stage to accomplish their cytoplasmic maturation; (d) whether the pre-GVBD RDC exists in mouse oocytes and its mechanism and significance; and (e) the discrepancy among reports on the effect of MAM with roscovitine on oocyte competence and the contradiction between chromatin condensation and gene transcription observed during MAM with roscovitine. The aim of the present study was to address these issues by examining the effects of MAM with roscovitine or db-cAMP on chromatin condensation, gene transcription and developmental potential in NSN or SN oocytes from prepubertal or adult mice.

## Results

### Classification of chromatin configuration in freshly collected prepubertal mouse oocytes

Chromatin configuration in freshly collected prepubertal mouse oocytes was classified into 4 patterns based on criteria reported previously^7^. In the NSN pattern, chromatin was the least condensed with sparse fine heterochromatin threads/granules that did not surround the nucleolus (Supplementary Fig. S1A). In the partial NSN (pNSN) pattern, some fine heterochromatin threads/granules began to appose to the nucleolus (Fig. S1B). In both the SN and the partial SN (pSN) oocytes, nucleoli were enclosed by heterochromatin. However, while the chromatin in SN oocytes was the most condensed with little diffuse chromatin (Fig. S1D), the chromatin in pSN oocytes was less condensed, always with some diffuse chromatin in the nucleoplasm (Fig. S1C). The proportions of NSN, pNSN, pSN and SN configurations were 44.6, 5.2, 10.3 and 39.9%, respectively, among the freshly collected oocytes (Fig. S1E). In the following experiments where oocytes with different configurations were cultured separately, the NSN and pNSN configurations were grouped together as NSN configuration, whereas the SN and pSN were put together as SN configuration, because it was difficult to distinguish between NSN and pNSN or between SN and pSN after the short Hoechst exposure for oocyte selection.

### Developmental competence of the NSN oocytes from prepubertal mice was severely impaired

**Figure 1:**
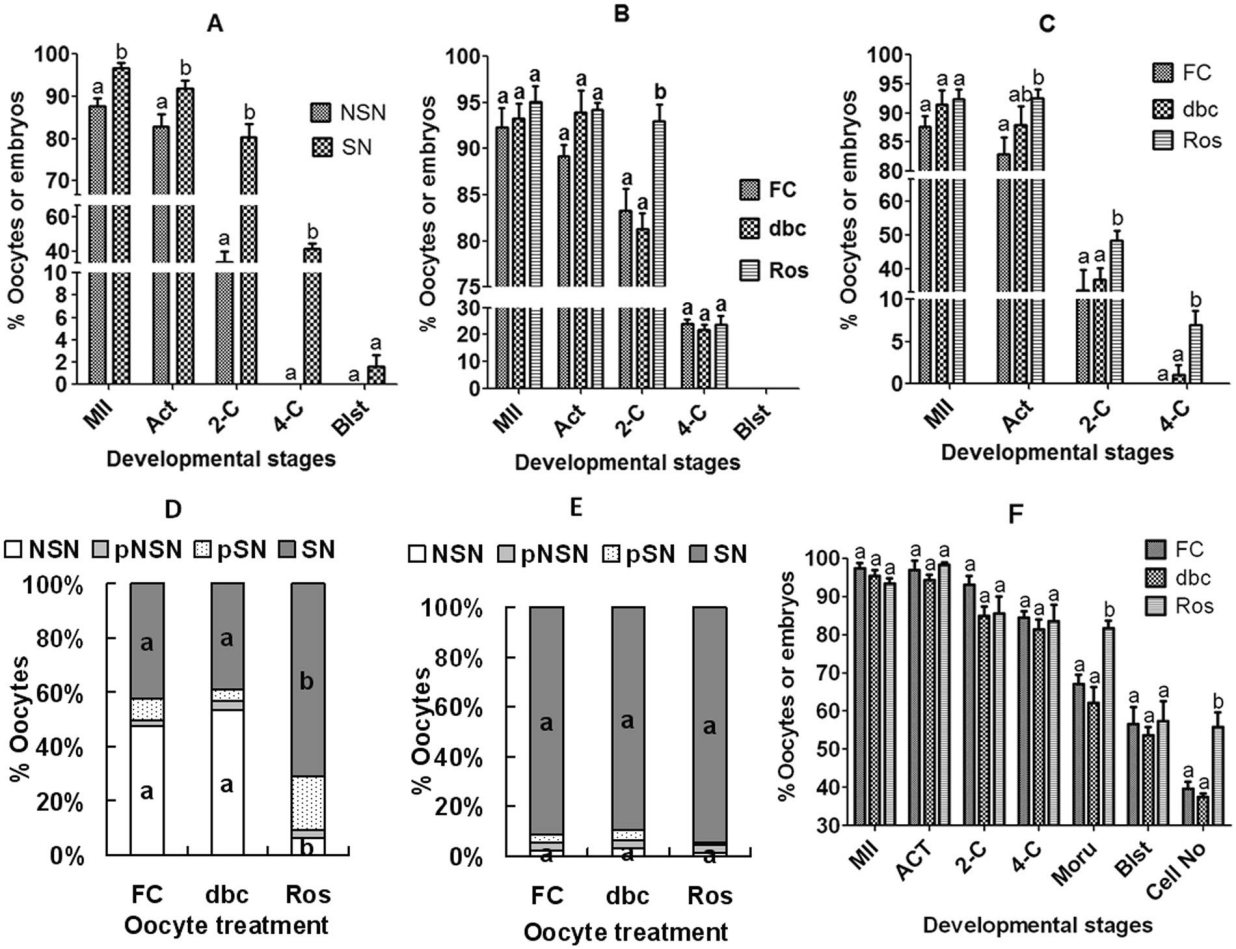
Effects of MAM with roscovitine or db-cAMP on maturation, embryo development and chromatin configuration of prepubertal (graphs **A** to **D**) and adult (graphs **E** and **F**) mouse oocytes. In graph **A**, freshly collected (FC) prepubertal oocytes were separated according to chromatin configuration and the resulting SN and NSN DOs were separately matured in 199-2 with cumulus monolayer coculture. In graph **B**, prepubertal COCs were matured in 199-2 following MAM for 4 h with 100 µM db-cAMP (dbc) or 100 µM roscovitine (Ros). In graph **C**, prepubertal DOs of NSN configuration were matured in 199-2 with cumulus monolayer following MAM for 4 h with dbc or Ros. Graphs **D** and **E** show distribution of different chromatin configurations following MAM of prepubertal and adult COCs, respectively, for 4 h with dbc or Ros. In graph **F**, adult COCs were blocked for 4 h in 199-1 containing dbc or Ros before *in vitro* maturation for embryo development. In graphs **A**,**B**,**C** and **F**, each treatment was repeated 6-7 times and each replicate contained about 35 oocytes. In graphs **D** and **E**, each treatment was repeated 4–5 times and each replicate contained about 20 oocytes. MII: % MII/cultured oocytes; Act: % Activated/MII oocytes; 2-C: % 2-cell embryos/activated oocytes; 4-C: % 4-cell embryos/2-C embryos; Blst: % Blastocysts/4-cell embryos. a,b: Values with a different letter above bars differ significantly (P < 0.05) within developmental stages or oocyte treatment groups.

Freshly-collected oocytes were separated according to chromatin configuration after Hoechst 33342 staining and the resulting DOs of SN and NSN patterns were matured separately. At 24 h of the maturation culture, oocytes were activated with SrCl_2_ for embryo development. Rates of maturation, activation and 2-cell embryos were all lower significantly in NSN oocytes than in SN oocytes, and no NSN oocytes developed to the 4-cell stage while 40% of the SN oocytes did (Fig. 1A). The results suggested that although the NSN oocytes were capable of nuclear maturation *in vitro*, their developmental competence was so severely impaired that many could not overcome the 2-cell block *in vitro*.

### Effects of MAM with roscovitine or db-cAMP on developmental potential of prepubertal mouse NSN and SN oocytes

Freshly collected COCs were blocked for 4 h in 199-1 containing db-cAMP or roscovitine. Oocytes were then matured for 24 h in 199-2 before SrCl_2_ activation for embryo development. Rates of oocyte maturation, activation and 4-cell embryos did not differ between treatments (Fig. 1B). However, the percentage of 2-cell embryos was significantly higher in roscovitine-blocked oocytes than in the db-cAMP blocked or non-blocked control oocytes. The results suggested that *in vitro* MAM of prepubertal mouse oocytes with roscovitine, but not with db-cAMP, facilitated the formation of 2-cell embryos.

To specify whether MAM with roscovitine improves competence of the NSN or SN oocytes, DOs of NSN and SN configuration were separately blocked with roscovitine or db-cAMP before maturation and activation for embryo development. Neither rates of maturation (ranging from 93% to 97%), activation (89% to 92%), 2-cell embryos (70% to 80%), 4-cell embryos (35% to 42%), nor rates of blastocysts (0% to 1.6%) differed significantly after SN oocytes were blocked with different regimens. When NSN oocytes were blocked, however, roscovitine significantly increased rates of activation, 2- and 4-cell embryos, whereas db-cAMP showed a limited effect (Fig. 1C). The results suggested that *in vitro* MAM of prepubertal oocytes with roscovitine mainly improved competence of NSN oocytes while having no effect on that of the SN oocytes.

### Effects of MAM with roscovitine or db-cAMP on chromatin configuration transition of prepubertal mouse oocytes

To test whether roscovitine promotes the NSN-SN transition, COCs were blocked for 4 h in 199-1 containing roscovitine or db-cAMP before observation for chromatin configurations. Compared to that observed in freshly collected oocytes, percentage of SN oocytes increased significantly after block with roscovitine, but it did not change following block with db-cAMP (Fig. 1D).

To confirm that roscovitine facilitate a NSN-SN transition while db-cAMP did not and to answer the question whether NSN oocytes have to go through a SN-stage before GVBD, live video microscopy was conducted to observe the pre-GVBD changes of chromatin configurations in DOs of NSN configuration during culture with or without roscovitine or db-cAMP. None of the 29 NSN oocytes cultured in 199-1 alone were found to take on the SN configuration before GVBD (Supplementary Fig. S2). While 28 out of the 31 oocytes cultured with roscovitine took on the SN configuration, 32 out of the 34 oocytes cultured with db-cAMP remained at the NSN stage. The results further confirm that NSN mouse oocytes undergo GVBD directly *in vitro* without going through SN, and that while roscovitine facilitated the NSN-SN transition, db-cAMP did not.

### Effects of MAM with roscovitine or db-cAMP on chromatin configuration and developmental potential of adult mouse oocytes

It has been reported that MAM with roscovitine promoted morula formation of oocytes from adult mice primed with eCG^31^, and that over 90% of these oocytes were of SN configuration^34^. Because this contradicts with the present results that meiotic arrest with roscovitine had no effect on SN oocytes of prepubertal mice, we reexamined the effects of MAM with roscovitine or db-cAMP on chromatin configuration and developmental competence of the adult mouse oocytes. COCs from adult mice were blocked for 4 h with roscovitine or db-cAMP before observation for chromatin configurations or *in vitro* maturation. Over 91% of the freshly recovered oocytes showed a SN configuration, and neither db-cAMP nor roscovitine treatment changed the proportion of oocytes with different chromatin configurations (Fig. 1E). Although rates of maturation, activation, 2-cell and 4-cell embryos and blastocysts did not differ among treatments, percentages of morulae and the cell number per blastocyst were significantly higher in roscovitine blocked oocytes than in freshly-collected control or db-cAMP-blocked oocytes (Fig. 1F). The results indicated that MAM with roscovitine increased the developmental potential of adult SN oocytes without changing their chromatin configuration, suggesting that chromatin condensation may not be an essential event for roscovitine to improve oocyte competence.

### Effects of MAM with roscovitine or db-cAMP on global gene transcription of prepubertal and adult mouse oocytes with different chromatin configurations

**Figure 2:**
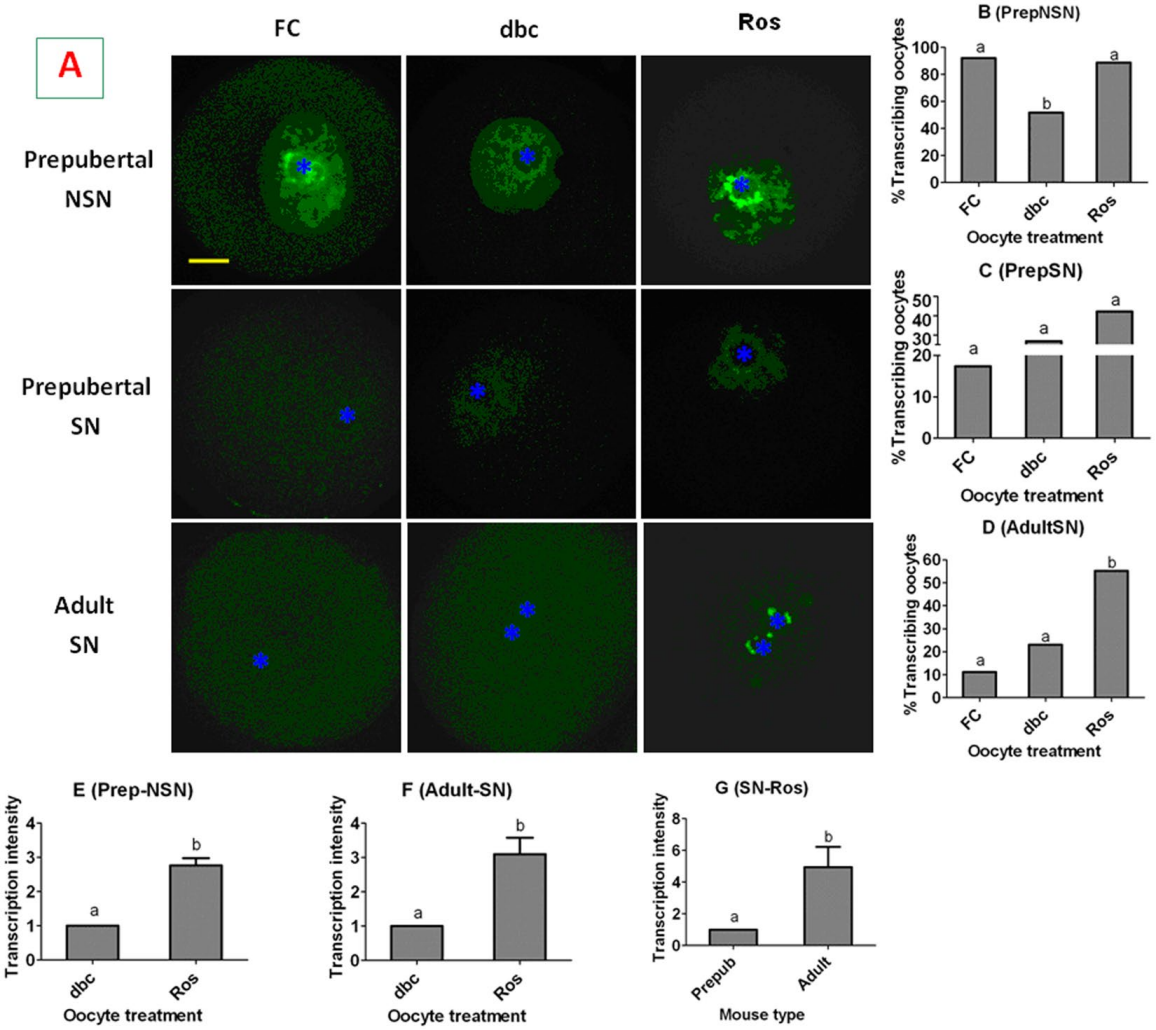
Global transcriptional activities in mouse oocytes. Freshly collected (FC) prepubertal or adult mouse DOs with different chromatin configurations were blocked for 4 h with 100 µM db-cAMP (dbc) or roscovitine (Ros). Panel A shows laser confocal images of FC or dbc- or Ros-blocked prepubertal or adult oocytes with NSN or SN configurations. The RNA was pseudo colored green. *Indicates the location of the nucleolus. The bar is 12 µm and applies to all images. Panels B,C and D show percentages of transcribing oocytes in prepubertal NSN, prepubertal SN and adult SN oocytes, respectively, immediately after collection or following MAM with dbc or Ros. Each treatment was repeated 3 times with each replicate containing 7 to 10 oocytes. Panels E and F show transcription (fluorescence) intensity in prepubertal NSN and adult SN oocytes, respectively, after MAM with dbc or Ros. Panel G compares transcription intensity between prepubertal and adult mice after MAM of SN oocytes with Ros. Each treatment was repeated 3 times and each replicate contained 5 most fluorescent oocytes selected from the treatment. a,b: Values with a different letter above bars differ significantly (P < 0.05).

Global gene transcription in prepubertal and adult mouse DOs with different chromatin configurations were observed immediately after recovery or following MAM with roscovitine or db-cAMP. About 90% of the freshly collected prepubertal NSN oocytes showed intensive transcriptional activities; although the percentage of transcribing oocytes decreased significantly following MAM with db-cAMP, it remained high after MAM with roscovitine (Fig. 2A and B). Few freshly-collected prepubertal SN oocytes show transcriptional activities, and the percentage of transcribing oocytes did not increase significantly following MAM with either db-cAMP or roscovitine (Fig. 2A and C). Even fewer freshly-collected adult SN oocytes show transcriptional activities; however, the percentage of transcribing oocytes increased significantly after MAM with roscovitine although it did not change following MAM with db-cAMP (Fig. 2A and D). Our analysis on transcriptional intensity further confirmed that the transcriptional activity in prepubertal NSN (Fig. 2E) and adult SN oocytes (Fig. 2F) was significantly higher after MAM with roscovitine than with db-cAMP, and the transcriptional intensity in adult SN oocytes was significantly higher than that in prepubertal SN oocytes following MAM with roscovitine (Fig. 2G). The results suggested that MAM with roscovitine promoted global gene transcription of both prepubertal NSN and adult SN oocytes but had no effect on that of prepubertal SN oocytes. MAM with db-cAMP facilitate transcription in none of the oocyte types tested.

**Figure 3:**
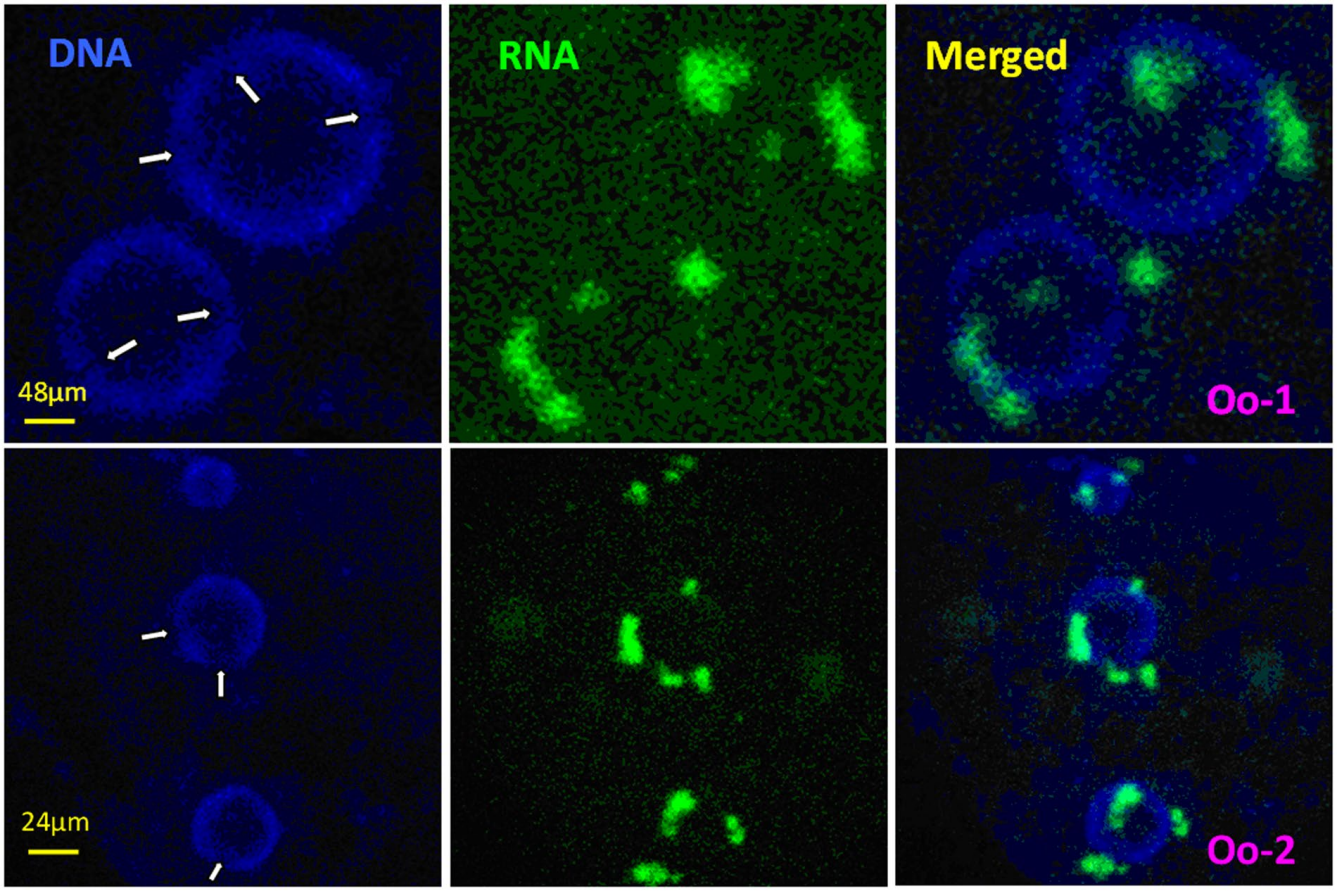
Enlarged confocal laser micrographs of adult SN oocytes 1 (Oo-1) and 2 (Oo-2) after MAM of DOs with 100 µM roscovitine showing the re-decondensed (RDC) sites (arrows) on the heterochromatin ring around the nucleolus and the sites of global gene transcription. Images in different columns from the left to right show chromatin (DNA), transcripts (RNA) and merged pictures, respectively.

In the above experiments, an intensive gene transcription was observed in both adult SN oocytes and the prepubertal NSN oocytes that had taken on a SN configuration following MAM with roscovitine. This apparently contradicts with the general rule that genes are silenced as chromatin condenses from the NSN to SN configuration. When confocal images of the roscovitine-treated SN oocytes were two- or four-fold enlarged and examined carefully, re-decondensed sites or notches with diffuse chromatin were observed on the heterochromatin ring around the nucleolus, and transcripts often clustered on or close to these notches (Fig. 3). It is thus suggested that roscovitine promoted gene transcription in the SN oocytes by inducing local RDC of chromatin on the heterochromatin shell around the nucleoli.

### Treatment with RNA polymerase II Inhibitor α-Amanitin abolished the beneficial effects of MAM with roscovitine on oocyte cytoplasmic maturation

**Figure 4:**
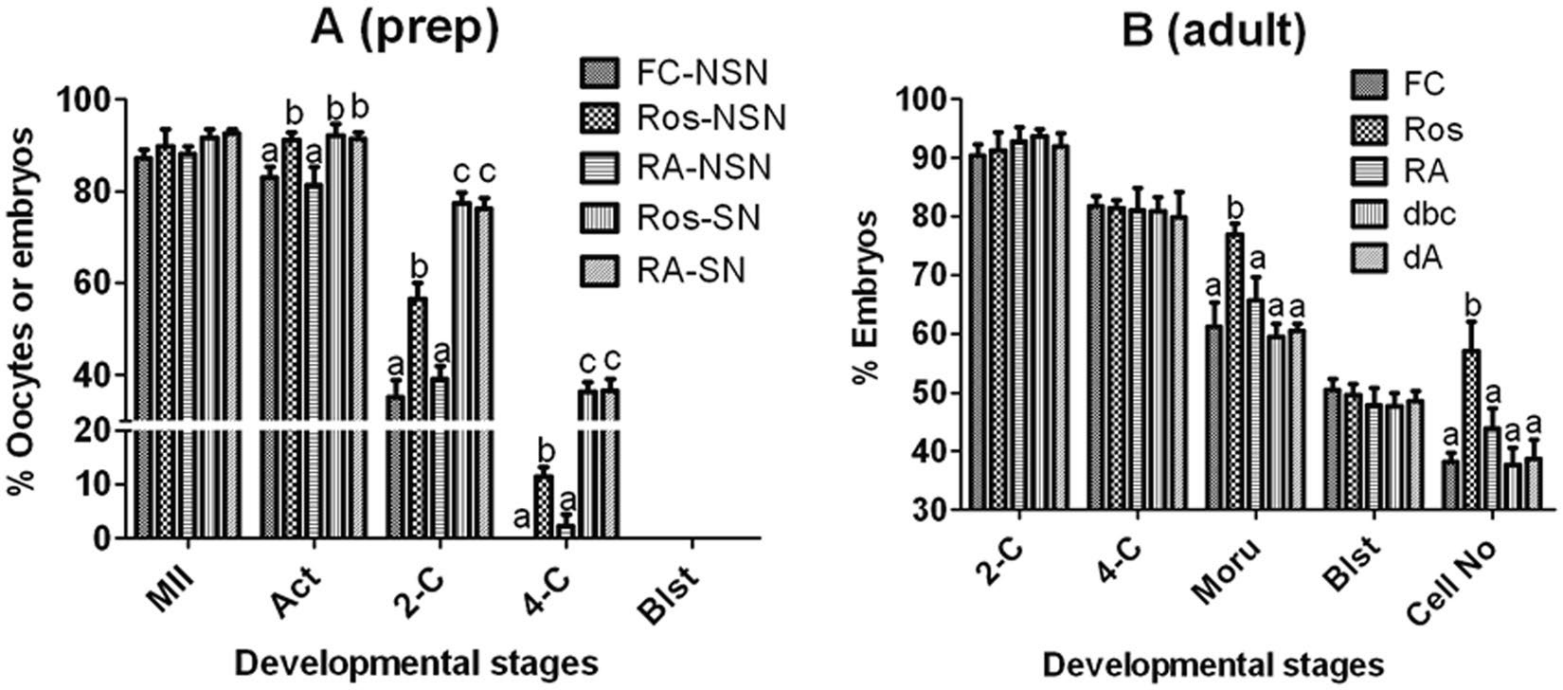
Effects of inhibiting RNA transcription with α-amanitin during MAM on oocyte developmental competence. NSN or SN DOs from prepubertal mice (graph **A**) or COCs from adult mice (graph **B**) were blocked for 4 h with 100 µM roscovitine (Ros) or db-cAMP (dbc) without or with 200 nM α-amanitin (RA or dA). At the end of MAM, oocytes were matured for 24 h before activation for embryo development. Freshly collected (FC) oocytes were matured without MAM to serve as controls. Each treatment was repeated 5 times and each replicate contained about 30 oocytes. MII: % MII/cultured oocytes; Act: % Activated/MII oocytes; 2-C: % 2-cell embryos/activated oocytes; 4-C: % 4-cell embryos/2-C embryos; Blst: % Blastocysts/4-cell embryos; Cell No: Cell number per blastocyst. a,b: Values with a different letter above bars differ significantly (P < 0.05) within developmental stages.

Prepubertal DOs with NSN or SN configuration and adult COCs were blocked for 4 h with roscovitine or db-cAMP without or with α-amanitin before maturation and activation for embryo development. Treatment with α-amanitin during MAM totally abolished the promoting effects of roscovitine on percentages of 2- and 4-cell embryos in prepubertal NSN oocytes (Fig. 4A) and on morula formation and cell number per blastocyst in adult oocytes (Fig. 4B). Treatment with α-amanitin during MAM did not affect the developmental potential of prepubertal SN oocytes blocked with roscovitine and that of adult oocytes blocked with db-cAMP, suggesting that the dosage of α-amanitin used was nontoxic to oocytes as both groups of oocytes were not transcribing. The results further confirmed that MAM with roscovitine improved oocyte competence by promoting gene transcription.

### MAM with roscovitine promoted gene transcription through inhibiting cyclin-dependent kinase (CDK) 5

**Figure 5:**
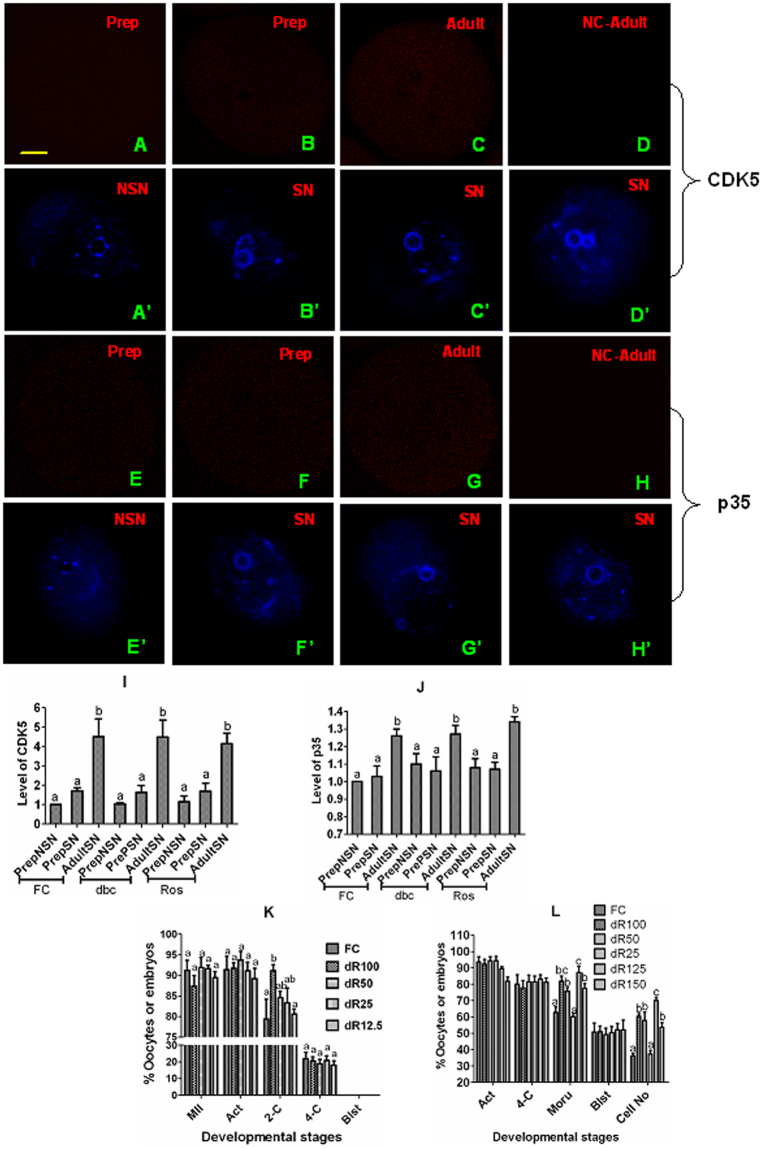
Contents and activity of CDK5 in mouse oocytes. (**A** to **J**) Show contents of CDK5 and p35 in NSN or SN oocytes from prepubertal (Prep) or adult mice before or after MAM of DOs with 100 µM db-cAMP (dbc) or roscovitine (Ros). Micrographs **A**/A’ to **H**/H’ are laser scanning confocal images. **A** and A’ etc. indicate the same oocytes observed using lasers and filters of different wave lengths following staining with anti-CDK5 or p35 antibodies (red) and Hoechst 33342 (blue), respectively. Bar is 12 µm and applies to all images. **D**/D’ and H/H’ show negative control (NC) oocytes processed with primary antibodies omitted. Graphs **I** and **J** show relative levels of CDK5 and p35, respectively, in prepubertal NSN or SN and adult SN oocytes before (FC) or after MAM with dbc or Ros. Each treatment was repeated 3 times and each replicate contained 10 oocytes. Graphs K and L show percentages of metaphase II (MII) and activated (Act) oocytes, 2-cell and 4-cell embryos and blastocyst (Blst) and cell number per blastocyst (Cell No) following prepubertal (**K**) or adult (**L**) COCs were blocked for 4 h with dbc and 100 (dR100), 50 (dR50), 25 (dR25), 12.5 (dR12.5), 125 (dR125) or 150 (dR150) µM Ros. Each treatment was repeated 3 times with each replicate containing about 30 oocytes. a,b: Values with a different letter above bars differ significantly (P < 0.05). In graph **L**, percentages of MII and activated oocytes, 2- and 4-cell embryos and blastocysts did not differ between treatments.

Why MAM with roscovitine promoted gene transcription and improved competence of adult SN oocytes but showed no effect on prepubertal SN oocytes? It is known that roscovitine inhibits a series of CDKs including CDK5^35^, and that CDK5 suppresses gene transcription through maintaining a high activity of HDAC^36^. We thus hypothesize that adult SN oocytes would contain more CDK5 than prepubertal SN oocytes, which makes the former more responsive to roscovitine to promote gene transcription than the latter. Contents of CDK5 and p35 were thus measured in NSN and SN oocytes from prepubertal or adult mice, as it has been reported that CDK5 is activated by two non-cyclin activators, p35 or p39^37^, and that both CDK5 and p35 are expressed in mouse ovaries including oocytes^38^. When observed under a laser confocal microscope after labeling with anti-CDK5 or -p35 antibodies, mouse oocytes showed fluorescence signals throughout the cytoplasm (Fig. 5A to H). When freshly collected oocytes were analyzed, the level of CDK5 in adult SN oocytes was 3 times higher than that in prepubertal NSN or SN oocytes (Fig. 5I). The level of p35 was also significantly higher in adult oocytes than in prepubertal oocytes (Fig. 5J). Both CDK5 and p35 levels remained unchanged after MAM of either prepubertal NSN or SN or adult SN oocytes with either db-cAMP or roscovitine. When prepubertal or adult COCs were blocked with different concentrations of roscovitine in the presence of db-cAMP, while 100 µM roscovitine significantly increased the percentage of 2-cell embryos in prepubertal COCs, and 100 or 125 µM roscovitine increased the percentage of morulae and cell counts of blastocysts in adult COCs, the effect decreased significantly with decreasing roscovitine concentrations (Fig. 5K and L). The results confirmed that adult SN oocytes were more responsive to roscovitine because they contained more CDK5/p35 than the prepubertal SN oocytes.

### MAM with roscovitine promoted chromatin condensation from NSN to SN through inhibiting CDK2

**Figure 6:**
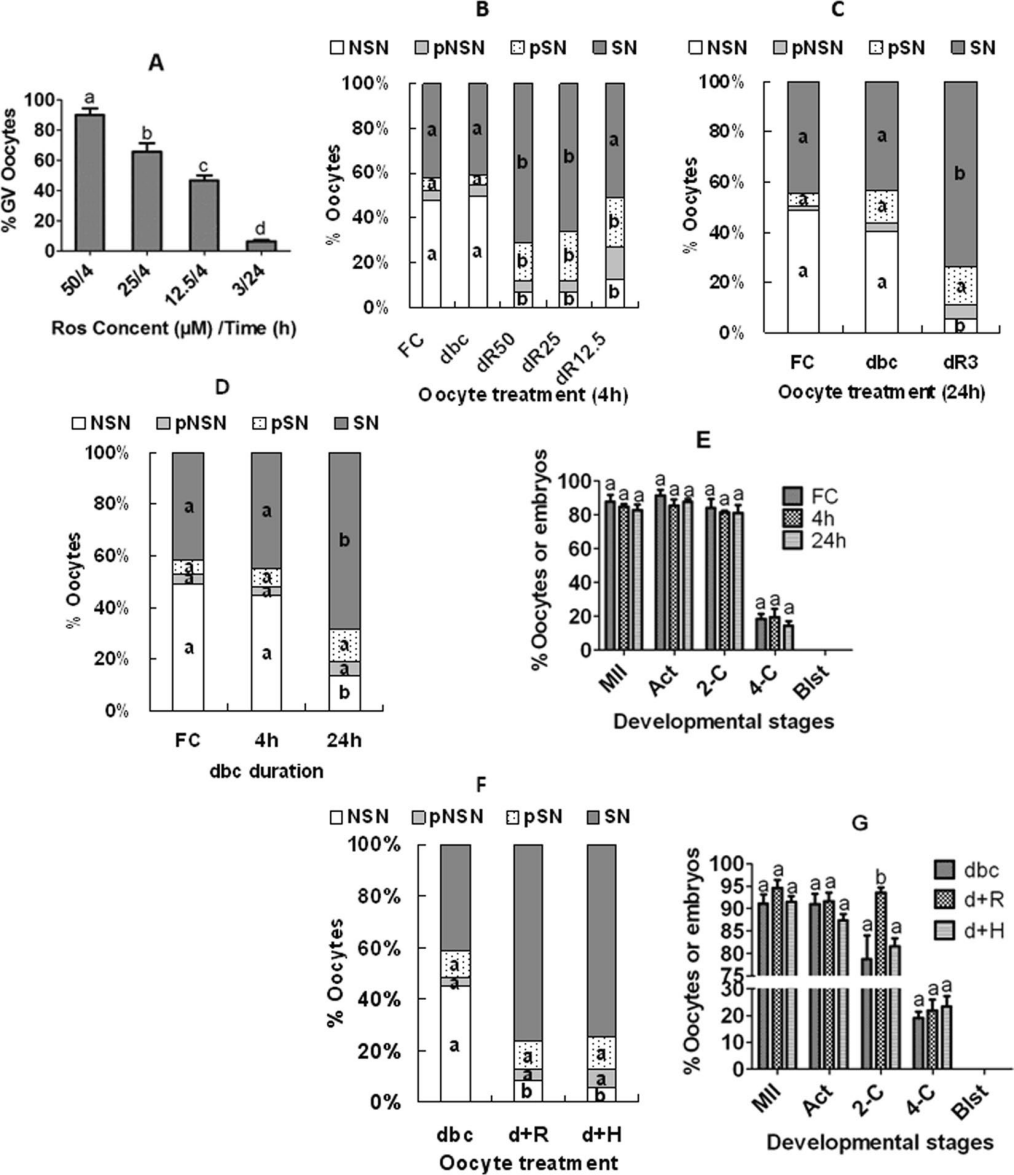
Effects of MAM of prepubertal COCs or DOs with roscovitine or H89 in the presence of db-cAMP on GVBD, chromatin configuration and developmental potential. Panel A shows percentages of GV-intact oocytes following MAM of COCs for 4 h with 50 (50/4), 25 (25/4) or 12.5 (12.5/4) µM roscovitine or for 24 h with 3 µM roscovitine (3/24). Panels B and C show percentages of oocytes with different chromatin configurations after MAM of COCs with db-cAMP and/or different concentrations of roscovitine for 4 and 24 h, respectively. Panels D and E show percentages of oocytes with different chromatin configurations and embryo development, respectively, after MAM of DOs with db-cAMP for different times. Panels F and G show percentages of oocytes with different chromatin configurations and embryo development, respectively, after MAM of COCs with roscovitine or H89 with or without db-cAMP for 12 h. FC: freshly collected oocytes; dbc: 100 µM db-cAMP alone; dR50, 25, 12.5 and 3: 100 µM db-cAMP plus 50, 25, 12.5 or 3 µM roscovitine, respectively; d+R and d+H: 100 µM db-cAMP plus 6 µM roscovitine or 5 µM H89, respectively; MII: metaphase II/cultured oocytes; Act: activated/MII oocytes; 2-C: 2-cell embryos/activated oocytes; 4-C: 4-cell/2-cell embryos; Blst: blastocyst /4-cell embryos. Each treatment was repeated 3–5 times with each replicate including about 30 oocytes. a-d: Values with a different letter above bars differ significantly (P < 0.05).

Both previous studies^27,32,33^ and the present results indicated that during MAM with roscovitine, chromatin condensed from NSN to SN configuration. How does roscovitine play the dual contradictory roles: while inhibiting chromosomal condensation and maintaining meiotic arrest on one hand, promoting chromatin condensation from NSN to SN configuration on the other hand? It has been reported that CDK1 is the only CDK essential and sufficient to drive meiosis resumption of mouse oocytes^39^, and that CDK2 is involved in histone H1 phosphorylation and large-scale chromatin decondensation in somatic cells^40^. We thus hypothesize that roscovitine might maintain meiotic arrest by inhibiting CDK1 while promoting chromatin condensation by suppressing CDK2, as it is known that roscovitine inhibits both CDK1 and CDK2^35^. To test this hypothesis, a series of roscovitine concentrations were tested using prepubertal mouse COCs to select one that could not inhibit CDK1 (GVBD) when used alone but could inhibit CDK2 (promote chromatin condensation) when used in combination with db-cAMP that does not promote chromatin condensation at all but maintain meiotic arrest when used alone. When MAM lasted for 4 h, while the percentage of GV-intact oocytes decreased significantly at 25 µM roscovitine (Fig. 6A), the percentage of SN oocytes increased significantly in the presence of both 25 µM roscovitine and db-cAMP compared to that after MAM with db-cAMP alone (Fig. 6B). When MAM lasted for 24 h, although GVBD was not inhibited at all at 3 µM roscovitine (Fig. 6A), the percentage of SN oocytes increased significantly at this roscovitine concentration in the presence of db-cAMP compared to that after MAM with db-cAMP alone (Fig. 6C). The results supported that roscovitine maintains meiotic arrest by inhibiting CDK1 but promotes chromatin condensation by suppressing CDK2. Thus, while lower concentrations of roscovitine could inhibit CDK2 (promote chromatin condensation), higher concentrations were required to inhibit CDK1 (GVBD).

### Extending the duration of MAM with db-cAMP induced chromatin condensation but had no effect on developmental competence of prepubertal DOs

The above results indicated that db-cAMP induced chromatin condensation in neither mouse COCs (Figs 1D and 6B,C) nor DOs (Fig. S2). However, Sun *et al*.^41^ showed in pig oocytes that db-cAMP promoted the NSN-SN transition in DOs while inhibiting it in COCs. Because it was reported that protein kinase A inhibited CDK2^42,43^, we suspected that the treatment duration (4 h) we used might not be long enough for db-cAMP to suppress CDK2 of mouse DOs. We thus extended the duration for db-cAMP treatment to see whether it would inhibit CDK2 of prepubertal DOs. Although the percentage of SN oocytes did not change after MAM with db-cAMP for 4 h, it increased significantly when the treatment was extended to 24 h (Fig. 6D). However, the developmental potential was not improved at all following MAM with db-cAMP for 24 h (Fig. 6E). The results confirmed that given enough time, db-cAMP could efficiently suppress CDK2 and induce chromatin condensation in mouse DOs but it could not improve their competence due to its inability to inhibit CDK5. In fact, it has been reported that PKA (cAMP) can activate CDK5^44–46^.

### H89, a PKA inhibitor, promoted chromatin condensation during MAM but did not improve developmental competence of prepubertal COCs

To verify whether the chromatin condensation induced by roscovitine is essential for improvement of oocyte competence, prepubertal COCs were blocked for 12 h with roscovitine or H89 in the presence of db-cAMP. Although both roscovitine and H89 promoted chromatin condensation (Fig. 6F), only roscovitine improved oocyte competence whereas H89 did not (Fig. 6G).

## Discussion

The present results showed that by using our optimized system for coculture of mouse DOs with cumulus cell monolayers^47^, over 85% of the NSN oocytes from prepubertal mice successfully matured *in vitro* following a classification under UV light after Hoechst 33342 staining. In early studies using simple culture conditions, however, only about 15% of the NSN oocytes isolated from adult mouse ovaries developed to the metaphase II stage after similar treatments of Hoechst staining and UV light classification^5,9,48^. Thus, the current results have unequivocally confirmed that the NSN mouse oocytes have the competence to complete nuclear maturation *in vitro*, given the optimal culture conditions.

The present results showed that while the developmental potential of both prepubertal NSN and adult SN oocytes was significantly improved, that of the prepubertal SN oocytes was not improved at all following MAM with roscovitine (Table S1). Our further observations with immunofluorescence microscopy and α-amanitin treatment indicated that MAM with roscovitine significantly promoted gene transcription in prepubertal NSN and adult SN oocytes, while it had no effect on that of the prepubertal SN oocytes. Although the percentage of oocytes with SN configuration increased significantly, their developmental competence was not improved at all following MAM of prepubertal DOs with db-cAMP for 24 h. While MAM with roscovitine significantly improved the developmental potential of adult oocytes, it had no effect on their chromatin configuration. Furthermore, while both db-cAMP+roscovitine and db-cAMP+H89 promoted chromatin condensation during MAM of prepubertal COCs, the latter did not improve their developmental competence while the former did. The results strongly suggested that it was gene transcription during maturation but not chromatin condensation (configuration transition) that determined the developmental potential of oocytes. By using a maternal restraint stress model and a mouse ovary-holding stress model, respectively, Wu *et al*.^34^ and Lin *et al*.^49^ observed that the developmental potential of SN mouse oocytes was more closely correlated with epigenetic histone modification than with chromatin configuration.

This study observed intensive gene transcription in prepubertal mouse NSN oocytes both before and at the end of MAM with roscovitine suggesting that gene transcription was uninterrupted in this group of oocytes during the 4 h of MAM with roscovitine. In the adult SN oocytes, however, gene transcription was obvious only after MAM with roscovitine and was always associated with re-decondensed (RDC) sites or notches of diffuse chromatin on the heterochromatin ring. Thus, while roscovitine promoted cytoplasmic maturation of the prepubertal NSN oocytes by maintaining a continuous gene transcription during the NSN-SN transition period, it did so in the adult SN oocytes by inducing a dotted RDC in the heterochromatin ring, which supported substantial gene transcription. An RDC with gene transcription has been observed in pig SN oocytes following MAM with roscovitine^22^, and gene transcription was evident in pre-GVBD oocytes of mice^16–19^ and humans^20,21^. However, the transcripts produced by the prepubertal NSN oocytes during the NSN-SN transition period might be different from those transcribed by the adult SN oocytes during RDC, as the formation of 2-cell embryos was improved in the former, whereas the formation of morulae was facilitated in the latter by MAM with roscovitine. Thus, it seems that while transcription during the NSN-SN transition is important for the first cleavage, transcription during RDC of the SN oocytes is essential for the morulae formation in mouse oocytes. Transcriptomic and proteomic profiling indicated that the 2-cell block in mouse NSN oocytes was associated with a lack of cytoplasmic lattices due to reduced expression of MATER and ribosomal proteins^50^. The beneficial effects of MAM with roscovitine on morula formation of the adult mouse oocytes have been reported^31^, but no reports have been published to date on its effects on the competence of prepubertal mouse oocytes.

It is well known that the developmental potential of oocytes from prepubertal animals is inferior to that of oocytes from adult animals^25,51,52^. However, the mechanisms for this age difference in oocyte competence are largely unknown. The present results revealed for the first time that MAM with roscovitine had differential effects on the developmental competence of prepubertal and adult mouse oocytes. For instance, MAM with roscovitine promoted gene transcription and increased morula formation of adult SN oocytes but had no effect on those of the prepubertal SN oocytes. Our further observations indicated that adult SN oocytes contained more CDK5/P35 and were thus more responsive to roscovitine to promote transcription than the prepubertal SN oocytes. Furthermore, compared to adult SN oocytes, the prepubertal SN oocytes lacked the ability to undergo RDC and failed to transcribe in response to the transcription/maturation stimulus. Taken together, the data suggested that during a regular *in vitro* maturation culture, the prepubertal NSN oocytes would undergo GVBD very quickly with no time to transcribe factors important for the first cleavage, and the prepubertal SN oocytes would be unable to transcribe factors essential for morula formation due to a lack of the RDC competence. Thus, the present results have provided new evidences to explain the mechanisms for the competence difference between prepubertal and adult oocytes.

In summary, the present results suggested that (a) there were intensive cellular activities such as gene transcription during MAM; (b) the NSN mouse oocytes have the competence to complete nuclear maturation *in vitro*, given the optimal culture conditions; (c) the difference in developmental competence between prepubertal NSN and SN oocytes was because whereas the former had not, the latter had completed the transcription of some important genes such as those essential for the first cleavage; (d) the difference in developmental potential between prepubertal and adult SN oocytes was because while the former had not, the latter had acquired the capacity for RDC and thus for transcription of important genes such as those essential for morula formation, suggesting that the SN chromatin configuration may not be an essential step for oocyte cytoplasmic maturation; and (e) roscovitine played dual contradictory roles during MAM: while facilitating chromatin condensation by inhibiting CDK2 on one hand, promoting gene transcription by suppressing CDK5 on the other hand. It is concluded that MAM with roscovitine improved oocyte competence by promoting gene transcription via inhibiting CDK5. Oocyte cytoplasmic maturation is correlated with gene transcription but not with chromatin condensation. The difference in developmental competence between prepubertal NSN and SN oocytes and between prepubertal and adult SN oocytes was because while the former had not, the latter had completed or acquired the ability for transcription of important genes.

## Methods

The experimental procedures were approved by the Animal Care and Use Committee of the Shandong Agricultural University P. R. China (Permit number: SDAUA-2001-0510). The methods were carried out according to the approved guidelines. Unless otherwise mentioned, all chemicals and reagents used in this study were purchased from Sigma Chemical Co. (St. Louis, MO, USA).

### Mice and oocyte collection

Mice of the Kunming strain were used in the present study. The mice were kept in a room under a 14L/10D photoperiod, with lights-off at 20:00. While prepubertal (18 to 19 days after birth) female mice were sacrificed without eCG-stimulation, adult (8 weeks after birth) female mice were sacrificed at 48 h following an intra-peritoneal injection of 10 IU equine chorionic gonadotropin (eCG, Ningbo Hormone Product company limited, China) to collect ovaries. After removing the surrounding adipose tissues and ovarian fallopian tubules, the large follicles on the ovary were ruptured in M2 medium to release cumulus-oocyte complexes (COCs). Only COCs with oocytes larger than 70 µm in diameter and showing a homogenous cytoplasm were selected for experiments.

### Chromatin configuration observation

The COCs were stripped of their cumulus cells by pipetting in M2 medium. For staining of fixed oocytes, the cumulus-denuded oocytes (DOs) were stained for 5 min with 10 µg/ml Hoechst 33342 contained in M2. The stained oocytes were then fixed in 4% paraformaldehyde for 40 min, mounted on a slide, and observed for GV chromatin configurations under a Leica DMLB fluorescence microscope. For staining of living oocytes, DOs were stained for 10 min in M2 medium containing 50 ng/ml Hoechst 33342. The stained oocytes were then placed in M2 medium containing 200 μM IBMX and examined for chromatin configurations under the Leica DMIRB fluorescence microscope. Then, oocytes with known chromatin configurations were washed in M2 medium and maturation medium, and cultured for maturation.

### Oocyte MAM culture

Oocyte MAM culture was performed in 100 µl microdrops (about 25 oocytes per drop) at 37 °C in a humidified atmosphere of 5% CO_2_ in air. The medium used for MAM was 199-1 supplemented with various concentrations of roscovitine or db-cAMP. The 199-1 medium was composed of the TCM-199 medium (Gibco, Grand Island, NY) supplemented with 10 IU/ml equine chorionic gonadotropin (eCG), 0.23 mM sodium pyruvate and 1 mg/ml bovine serum albumin (BSA, Gibco). To prepare stock solutions, roscovitine (50 mM) and H89 (5 mM) were dissolved in dimethyl sulfoxide (DMSO, Gibco), and db-cAMP (200 mM) was dissolved in water. The stock solutions were stored in aliquots at −20 °C until use.

### Oocyte maturation ***in vitro***

#### Preparation of cumulus cell monolayers

Around 20 oocytes obtained from adult mice at 48 h after eCG injection were washed in M2 medium and repeatedly pipetted in DMEM medium (Gibco) to recover cumulus cells. The collected cumulus cells were then washed three times in DMEM medium, transferred into wells of a 96-well plate together with 150 µl DMEM medium and incubated at 37 °C in humidified atmosphere containing 5% CO_2_ in air. When the cumulus cells grew to 80% of confluence, the spent medium in the wells of the 96-well culture plate was replaced with 100 µl oocyte maturation medium and equilibrated for 3 h in a CO_2_ incubator before use for oocyte maturation culture.

#### Oocyte maturation culture

About 25 oocytes were cultured in 100 µl drops at at 37 °C in a humidified atmosphere of 5% CO_2_ in air. The maturation medium used was 199-2, which consisted of TCM-199 supplemented with 10% (v/v) BSA, 1 µg/ml 17β-estradiol, 24.2 mg/ml sodium pyruvate, 0.05 IU/ml follicle stimulating hormone (FSH), 0.05 IU/ml luteinizing hormone (LH), and 10 ng/ml epidermal growth factor (EGF). While COCs were cultured directly in 199-2, DOs were cultured on cumulus cell monolayers in 199-2 supplemented with 200 µM cystine and 400 µM cysteamine.

### Oocyte activation and embryo culture

At 24 h of maturation culture, while DOs were treated directly, COCs were stripped of cumulus cells by pipetting in M2 containing 0.1% hyaluronidase. After being washed twice in M2 and once in the activating medium, the oocytes were incubated in an activating medium for 6 h at 37 °C in a humidified atmosphere with 5% CO_2_ in air. The activating medium used was Ca^2+^-free CZB medium supplemented with 10 mM SrCl_2_ and 5 µg/ml cytochalasin B. At the end of the activating treatment, the oocytes were examined for activation under a phase contrast microscope. Oocytes were considered activated when each contained one or two well-developed pronuclei. Activated oocytes were cultured for 4 days in regular CZB (30-35 oocytes per 100 µl drop) at 37 °C under humidified atmosphere with 5% CO_2_ in air. Glucose (5.5 mM) was added to CZB when embryos developed beyond 3- or 4-cell stages. Embryos were examined at 24 h, 48 h, 72 h and 96 h after activation treatment to record the numbers of 2-cell, 4-cell embryos, morulae and blastocysts, respectively. To calculate cell number per blastocyst, all the blastocysts in each replicate were stained with 10 µg/ml Hoechst 33342 before cell counting.

### Time-lapse imaging

Prepubertal DOs were stained for 10 min in M2 medium containing 50 ng/ml Hoechst 33342. Then, the selected NSN-oocytes were transferred to and cultured in 199-1 containing 20 ng/ml Hoechst 33342 in an incubator (INUBG2E-ZILCS; Tokai Hit) and observed under a video microscope (DMI6000B; Leica). Phase contrast and fluorescence images of oocytes were taken at 5-min intervals with a charge-coupled device camera (885 EM; Andor), and the chromatin was pseudo-colored red.

### Detection of global RNA transcription

Oocytes were labeled for 1 h in 100 µl 199-1 containing 1 mM 5-ethynyl uridine (EU). All the procedures for EU detection were conducted at room temperature in accordance with the manufacturer’s protocols (Invitrogen; Click-iT RNA imaging kits). After EU labeling, oocytes were (1) fixed with 3.7% formaldehyde in PBS for 40 min, (2) permeabilized with 0.1% Triton X-100 for 30 min, (3) stained in the dark for 30 min with 100 mM Tris (pH 8.5)/1 mM CuSO_4_/10–50 µM fluorescent azide/100 mM ascorbic acid, (4) washed with Click-iT® reaction rinse buffer, (5) stained with Hoechst 33342 and (6) mounted on glass slides and observed with a Leica laser scanning confocal microscope (TCS SP2; Leica Microsystems). Blue diode (405 nm) and argon (Ar; 488 nm) lasers were used to excite Hoechst and FITC, respectively. Fluorescence of Hoechst and FITC were detected with 420–480 nm and 505–540 nm bandpass emission filters, respectively.

### Immunofluorescence for expression detection of key proteins

The DOs were (1) fixed for 1 h with 4% paraformaldehyde, (2) permeabilized with 0.5% Triton X-100 for 15 min, (3) blocked for 1 h in regular PBS containing 3% BSA and 0.1% Tween-20, (4) incubated at 4 °C overnight with Anti-p35 (1:50, Beyotime, AP035) or Anti-Cdk5[EP715Y] (1:200, Abcam, ab40773), (5) incubated at room temperature for 1 h with CyTM3-conjugated AffiniPure Goat Anti-Rabbit IgG (H+L) (1:800 dilution, Jackson ImmunoRerearsh, 111–165–144), (6) stained for 5 min with 10 µg/ml Hoechst 33342 to observe chromatin, and (7) mounted on a glass slide and observed with a laser confocal microscope (TCS SP2; Leica Microsystems). Blue diode (405 nm) and helium/neon (He/Ne; 543 nm) lasers were used to excite Hoechst and CyTM3, respectively. Fluorescence of Hoechst and CyTM3 was detected with 420–480 nm and 550–570 nm bandpass emission filters, respectively, and the captured signals were recorded as blue and red, respectively.

The relative levels of each protein expression were quantified by measuring fluorescence intensities. The fluorescence intensities were measured on the raw images using the Image-Pro Plus software (Media Cybernetics Inc., Silver Spring, MD) under fixed thresholds across all the slides. Both the fluorescence density and the area of the objects giving the fluorescence were measured and the mean relative intensity of fluorescence was calculated for each oocyte. For each protein, the average relative fluorescence of the NSN oocytes was set to one and that of oocytes from other treatments were expressed relative to this value. Several measures were taken to ensure the accuracy of protein quantification. Firstly, oocytes used for one or two replicates of all treatments in the same experiment were processed and observed on the same day. Secondly, when mounting oocytes onto slides, care was taken to ensure that oocytes on different slides were compressed to the same extent and had a similar thickness. Thirdly, for each experimental series, all the images were acquired with identical settings. Fourthly, within each oocyte, a single plane with maximum amount of chromatin and maximum fluorescence intensity was selected to take photograph for further analysis.

### Data analysis

Each treatment contained at least three replicates. Percentage data were arc sine transformed before analysis. Data were analyzed with ANOVA when each measure contained three or more groups. A Duncan multiple comparison test was conducted to found differences. Data were analyzed using an independent t-test when each measure contained only two groups. The software used was Statistics Package for Social Sciences (version 11.5; SPSS, Inc.). Data were expressed as means ± SEM, and P < 0.05 was considered significant.

## Electronic supplementary material

Supplementary information

## References

[1] Hyttel P, Fair T, Callesen H, Greve T (1997). Oocyte growth, capacitation and final maturation in cattle. Theriogenology..

[2] Gosden R, Krapez J, Briggs D (1997). Growth and development of the mammalian oocyte. Bioessays..

[3] Romero S, Sánchez F, Lolicato F, Van Ranst H, Smitz J (2016). Immature oocytes from unprimed juvenile mice become a valuable source for embryo production when using C-type natriuretic peptide as essential component of culture medium. Biol Reprod..

[4] Wickramasinghe D, Ebert KM, Albertini DF (1991). Meiotic competence acquisition is associated with the appearance of M-phase characteristics in growing mouse oocytes. Dev Biol..

[5] Debey P (1993). Competent mouse oocytes isolated from antral follicles exhibit different chromatin organization and follow different maturation dynamics. Mol Reprod Dev..

[6] Fuente DL (2006). R. Chromatin modifications in the germinal vesicle (GV) of mammalian oocytes. Dev Biol..

[7] Tan JH (2009). Chromatin configurations in the germinal vesicle of mammalian oocytes. Mol Hum Reprod..

[8] Zuccotti M (1998). Meiotic and developmental competence of mouse antral oocytes. Biol Reprod..

[9] Zuccotti M (2002). The analysis of chromatin organisation allows selection of mouse antral oocytes competent for development to blastocyst. Zygote..

[10] Zuccotti M, Garagna S, Merico V, Monti M, Alberto Redi C (2005). Chromatin organisation and nuclear architecture in growing mouse oocytes. Mol Cell Endocrinol..

[11] Parfenov V, Potchukalina G, Dudina L, Kostyuchek D, Gruzova M (1989). Human antral follicles: oocyte nucleus and the karyosphere formation (electron microscopic and autoradiographic data). Gamete Res..

[12] Miyara F (2003). Chromatin configuration and transcriptional control in human and mouse oocytes. Mol Reprod Dev..

[13] Liu Y (2006). Germinal vesicle chromatin configurations of bovine oocytes. Microsc Res Tech..

[14] Hunter AG, Moor RM (1987). Stage-dependent effects of inhibiting ribonucleic acids and protein synthesis on meiotic maturation of bovine oocytes *in vitro*. J. Dairy Sci..

[15] Kastrop PM, Hulshof SC, Bevers MM, Destree OH, Kruip TA (1991). The effects of a-amanitin and cycloheximide on nuclear progression, protein synthesis, and phosphorylation during bovine oocyte maturation *in vitro*. Mol Reprod Dev..

[16] Bloom AM, Mukherjee BB (1972). RNA synthesis in maturing mouse oocytes. Exp Cell Res..

[17] Rodman TC, Bachvarova R (1976). RNA synthesis in preovulatory mouse oocytes. J. Cell Biol..

[18] Wassarman PM, Letourneau GE (1976). RNA synthesis in fully-grown mouse oocytes. Nature..

[19] Kopecny V, Landa V, Pavlok A (1995). Localization of nucleic acids in the nucleoli of oocytes and early embryos of mouse and hamster: An autoradiographic study. Mol Reprod Dev..

[20] Tesarik J, Travnic P, Kopecny V, Kristek F (1983). Nucleolar transformations in the human oocyte after completion of growth. Gamete Res..

[21] Tesarik J, Kopecny V, Kurilo LF (1984). Pre-ovulatory RNA synthesis in human oocytes of large antral follicles. Histochem J..

[22] Zhang M (2017). Meiotic arrest with roscovitine and follicular fluid improves cytoplasmic maturation of porcine oocytes by promoting chromatin de-condensation and gene transcription. Sci Rep..

[23] Ledda S, Bogliolo L, Calvia P, Leoni G, Naitana S (1997). Meiotic progression and developmental competence of oocytes collected from juvenile and adult ewes. J. Reprod Fertil..

[24] Marchal R (2001). Meiotic and developmental competence of prepubertal and adult swine oocytes. Theriogenology..

[25] Jiao GZ (2013). Developmental potential of prepubertal mouse oocytes is compromised due mainly to their impaired synthesis of glutathione. PLoS One..

[26] Mermillod P, Tomanek M, Marchal R, Meijer L (2000). High developmental competence of cattle oocytes maintained at the germinal vesicle stage for 24 hours in culture by specific inhibition of MPF kinase activity. Mol Reprod Dev..

[27] Han D (2006). Factors affecting the efficiency and reversibility of roscovitine (ROS) block on the meiotic resumption of goat oocytes. Mol Reprod Dev..

[28] Crocomo LF (2016). *In vitro* developmental competence of adult sheep oocytes treated with roscovitine. Reprod Domest Anim..

[29] Romar R, Funahashi H (2006). *In vitro* maturation and fertilization of porcine oocytes after a 48 h culture in roscovitine, an inhibitor of p34cdc2/cyclin B kinase. Anim Reprod Sci..

[30] Sananmuang T, Techakumphu M, Tharasanit T (2010). The effects of roscovitine on cumulus cell apoptosis and the developmental competence of domestic cat oocytes. Theriogenology..

[31] Sanfins A, Plancha CE, Albertini DF (2015). Pre-implantation developmental potential from *in vivo* and *in vitro* matured mouse oocytes: a cytoskeletal perspective on oocyte quality. J. Assist Reprod Genet..

[32] Kubelka M, Motlík J, Schultz RM, Pavlok A (2000). Butyrolactone I reversibly inhibits meiotic maturation of bovine oocytes, without influencing chromosome condensation activity. Biol Reprod..

[33] Kubelka M, Anger M, Kalous J, Schultz RM, Motlík J (2002). Chromosome condensation in pig oocytes: lack of a requirement for either cdc2 kinase or MAP kinase activity. Mol Reprod Dev..

[34] Wu XF (2015). Restraint stress on female mice diminishes the developmental potential of oocytes: roles of chromatin configuration and histone modification in germinal vesicle stage oocytes. Biol Reprod..

[35] Meijer L (1997). Biochemical and cellular effects of roscovitine, a potent and selective inhibitor of the cyclin-dependent kinasescdc2, cdk2 and cdk5. Eur J. Biochem..

[36] Li Z (2004). Cdk5/p35 phosphorylates mSds3 and regulates mSds3-mediated repression of transcription. J. Biol Chem..

[37] Dhavan R, Tsai LH (2001). A decade of CDK5. Nat Rev Mol Cell Biol..

[38] Lee KY (2004). Cdk5/p35 expression in the mouse ovary. Mol Cells..

[39] Adhikari D (2012). Cdk1, but not Cdk2, is the sole Cdk that is essential and sufficient to drive resumption of meiosis in mouse oocytes. Hum Mol Genet..

[40] Alexandrow MG, Hamlin JL (2005). Chromatin decondensation in S-phase involves recruitment of Cdk2 by Cdc45 and histone H1 phosphorylation. J. Cell Biol..

[41] Sun MJ (2016). An essential role for the intra-oocyte MAPK activity in the NSN-to-SN transition of germinal vesicle chromatin configuration in porcine oocytes. Sci Rep..

[42] D’Angiolella V (2001). Role for cyclin-dependent kinase 2 in mitosis exit. Curr Biol..

[43] Brenner AK, Reikvam H, Lavecchia A, Bruserud Ø (2014). Therapeutic targeting the cell division cycle 25 (CDC25) phosphatases in human acute myeloid leukemia–the possibility to target several kinases through inhibition of the various CDC25 isoforms. Molecules..

[44] Lin H, Chen MC, Ku CT (2009). Cyclin-dependent kinase 5 regulates steroidogenic acute regulatory protein and androgen production in mouse Leydig cells. Endocrinology..

[45] Chen MC (2010). Involvement of cAMP in nerve growth factor-triggered p35/Cdk5 activation and differentiation in PC12 cells. Am J. Physiol Cell Physiol..

[46] Jeon S, Kim Y, Chung IW, Kim YS (2015). Clozapine induces chloride channel-4 expression through PKA activation and modulates CDK5 expression in SH-SY5Y and U87 cells. Prog Neuropsychopharmacol Biol Psychiatry..

[47] Zhou P (2010). Mouse cumulus-denuded oocytes restore developmental capacity completely when matured with optimal supplementation of cysteamine, cystine, and cumulus cells. Biol Reprod..

[48] Ola SI (2007). Meiotic competence and acetylation pattern of UV light classified mouse antral oocytes after meiotic arrest with isobutylmethylxanthine. Mol Reprod Dev..

[49] Lin J (2016). The relationship between apoptosis, chromatin configuration, histone modification and competence of oocytes: A study using the mouse ovary-holding stress model. Sci Rep..

[50] Monti M (2013). Developmental arrest and mouse antral not-surrounded nucleolus oocytes. Biol Reprod..

[51] Revel F, Mermillod P, Peynot N, Renard JP, Heyman Y (1995). Low developmental capacity of *in vitro* matured and fertilized oocytes from calves compared with that of cows. J. Reprod Fertil..

[52] O’Brien JK, Catt SL, Ireland KA, Maxwell WM, Evans G (1997). *In vitro* and *in vivo* developmental capacity of oocytes from prepubertal and adult sheep. Theriogenology..

