# Supplementary material for: Mechanisms by which in vitro meiotic arrest and sexual maturity improve developmental potential of mouse oocytes
Source: Sci Rep. 2017 Nov 17;7:15763. doi: 10.1038/s41598-017-16119-5 (PMC5693946; doi:10.1038/s41598-017-16119-5)
Supplement: Supplementary file 1 — Supplementary information [file 41598_2017_16119_MOESM1_ESM.doc]

Supplementary information

**Title: Mechanisms by which in vitro meiotic arrest and sexual maturity improve developmental potential of mouse oocytes**

Fei Chen^1^, Juan Lin^2^, Xue Sun^2^, Bin Xiao^2^, Shu-Fen Ning^2^, Shuai Zhu^2^, Hui-Li Wang^2^ and Jing-He Tan^1,2,3^

1. College of Life Science, Northeast Agricultural University, Harbin, 150030, P. R. China

2. College of Animal Science and Veterinary Medicine, Shandong Agricultural University, Tai-an City 271018, P. R. China

3. Corresponding author: Jing-He Tan, College of Animal Science and Veterinary Medicine, Shandong Agricultural University, Tai-an City, Shandong Province, P R China

Post code: 271018; Phone: 0538-8249616; FAX: 0538-8241419; Email: tanjh@sdau.edu.cn

Supplementary figure and table legends

Supplementary Figure S1. Classification of chromatin configurations in freshly collected prepubertal mouse oocytes.

Supplementary Figure S2. Live video microscopic observation on pre-GVBD changes of chromatin configurations in mouse DOs of NSN configuration when cultured in 199-1 alone (Control), or with roscovitine or db-cAMP. Images (left to right) were taken every 5 min in control oocytes (n=29) but every 10 min in roscovitine (n=31) or db-cAMP (n=34) treated oocytes. Original magnification ×100.

Supplementary Table S1. A summary of the intra-oocyte events during MAM with roscovitine or db-cAMP in oocytes with NSN or SN configurations from prepubertal or adult mice.


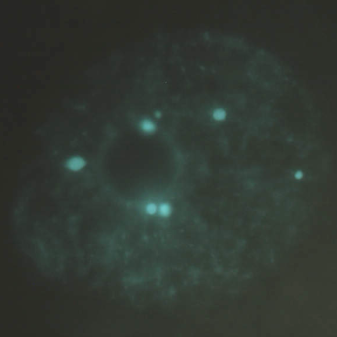


*****

**pNSN**

**B**


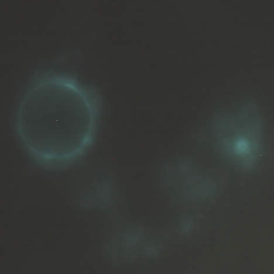


*****

**D**

**SN**


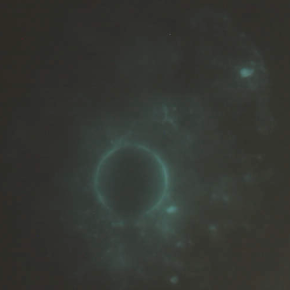


*****

**pSN**

**C**


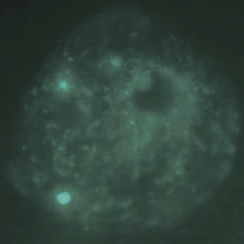


*****

**NSN**

**A**

Supplementary Figure S1. Classification of chromatin configurations in freshly collected prepubertal mouse oocytes.


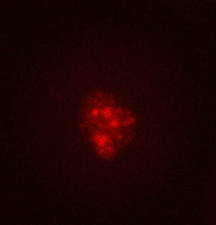


**0 min**


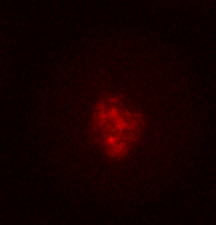


**15 min**


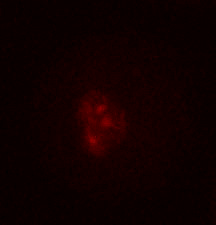


**30 min**


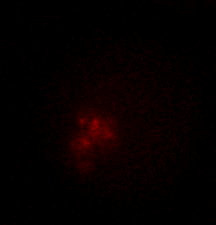


**45 min**

**min**


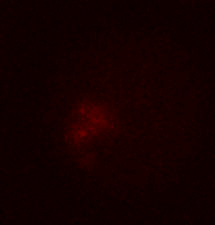


**50 min**


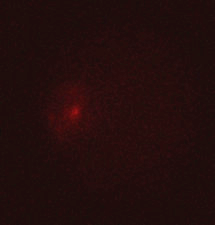


**55 min**


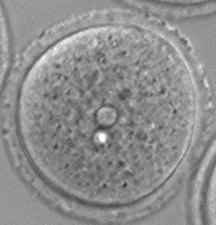

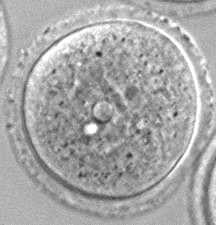

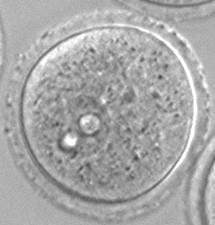

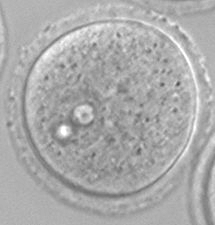

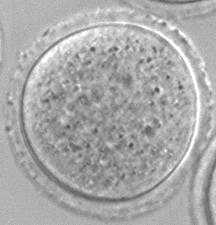

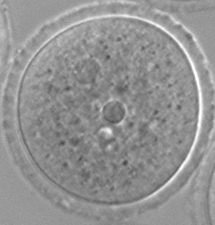


**Control**


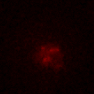


**0 min**


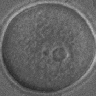

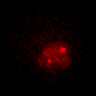


**60 min**


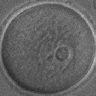

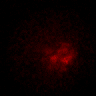


**120 min**


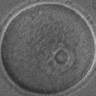

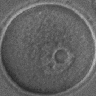

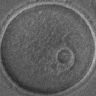

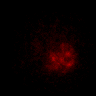


**150 min**


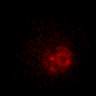


**180 min**


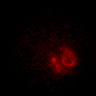


**210 min**


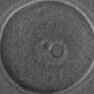


**Roscovitine**


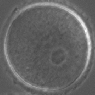

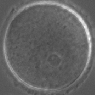

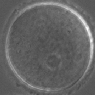

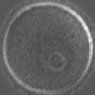

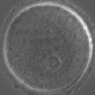

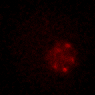


**0 min**


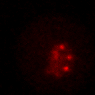


**60 min**


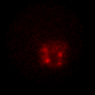


**120 min**


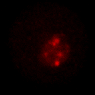


**150 min**


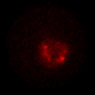


**180 min**


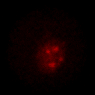


**210 min**


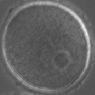


**db-cAMP**

Supplementary Figure S2. Live video microscopic observation on pre-GVBD changes of chromatin configurations in mouse DOs of NSN configuration when cultured in 199-1 alone (Control), or with roscovitine or db-cAMP. Images (left to right) were taken every 5 min in control oocytes (n=29) but every 10 min in roscovitine (n=31) or db-cAMP (n=34) treated oocytes. Original magnification ×100.

Supplementary Table S1. A summary of the intra-oocyte events during MAM with roscovitine or db-cAMP in oocytes with NSN or SN configurations from prepubertal or adult mice^*^

| MAM with | Oocyte type | CDK1 | CDK2 | CDK5 | Develop potential | Chromatin condense | Gene transcript |
| --- | --- | --- | --- | --- | --- | --- | --- |
| Roscovitine | Prep-NSN | ↓ | ↓ | ↓ | ↑ | ↑ | ↑ |
|  | Prep-SN | ↓ | ↓ | ↓ | − | − | − |
|  | Adult-SN | ↓ | ↓ | ↓ | ↑ | − | ↑ |
| db-cAMP | Prep-NSN | ↓ | ↓ | ↑ | − | ↑ | − |
|  | Prep-SN | ↓ | ↓ | ↑ | − | − | − |
|  | Adult-SN | ↓ | ↓ | ↑ | − | − | − |

^*^ When CDK1, CDK2 and CDK5 were all inhibited during MAM with roscovitine, both developmental potential and gene transcription in prepubertal NSN and adult SN oocytes increased significantly but those in the prepubertal SN oocytes did not change at all. When CDK1 and CDK2 were inhibited while CDK5 was activated during MAM with db-cAMP, however, no increase in either developmental potential or gene transcription was observed in any of the three types of oocytes. Chromatin condensation from NSN to SN configuration was obvious in the prepubertal NSN oocytes during MAM when CDK2 was inhibited with either roscovitine or db-cAMP.
